# Supplementary material for: Omics Approaches in Uncovering Molecular Evolution and Physiology of Botanical Carnivory
Source: Plants (Basel). 2023 Jan 15;12(2):408. doi: 10.3390/plants12020408 (PMC9867145; doi:10.3390/plants12020408)
Supplement: Supplementary file 1 [file plants-12-00408-s001.zip › plants-2092953-supplementary.pdf]

Supplementary Table S1. A summary of genomic and transcriptomic studies on carnivorous plant species.

Species	Genome size (Mbp)	Plastome size (kbp)	Sequencing platform(s)	Genome (BioProject)	Plastome (NCBI RefSeq)	Transcriptome (BioProject)	References
A. vesiculosa	509	141.6	PacBio RS, Illumina HiSeq	PRJEB35196 PRJNA394436 PRJNA453847	NC_035416.1	-	[11]
C. follicularis	2,110	142.7	Illumina HiSeq; Illumina MiSeq	PRJDB4484 PRJNA548736 PRJNA810039 (metagenome)	NC_042597.1	PRJDB4484	[41,63]
D. binata, D. lusitanicum, N. alata	-	-	-	-	-	PRJNA350559	[2]
D. rotundifolia	-	192.9	Roche 454 GS-FLX; Illumina MiSeq	PRJNA316228	NC_029770.1	PRJNA534215	[19]
D. muscipula	3,180	117.6	PacBio RS; Illumina HiSeq; Illumina MiSeq	PRJEB35195 PRJNA394358 PRJNA453821 PRJNA790936 PRJEB15538 (metagenome)	NC_035417.1	PRJEB38423 PRJNA203407 PRJNA208984 PRJNA530242 PRJNA862127	[11-14]
D. capensis	293	-	Illumina HiSeq	PRJNA291419	-	-	[17]
D. spatulata	323	-	PacBio RS II; Illumina HiSeq	PRJDB9009 PRJNA790939	-	PRJDB9009	[11]
G. aurea	63.6	140.0	Illumina HiSeq	PRJNA208769 PRJNA438327	NC_037078.1	-	[7,64]
N. ampullaria	-	-	PacBio RS II; Illumina HiSeq	-	-	PRJNA279267 PRJNA299862	[32,34,37,65]
N. khasiana	-	156.9	Illumina HiSeq	PRJNA680098 PRJNA473234	NC_051455.1	PRJEB22838 PRJNA296942 PRJNA345154	[30,66]
N. mirabilis	691.4	155.8	Roche 454 GS-FLX; DNBSEQ-T7	PRJNA529137 PRJNA869487	NC_041271.1	PRJNA387677 PRJNA883181	[24,67]

N. rafflesiana	-	-	PacBio RS II;<br>Illumina HiSeq	-	-	PRJNA282399<br>PRJNA299862	[23,32,37]
N. × *hookeriana* (*N. rafflesiana* × *N. ampullaria*)	-	-	PacBio RS II	-	-	PRJNA299862	[32,37]
N. × *ventrata* (*N. alata* × *N. ventricosa*)	-	156.6	Illumina HiSeq;<br>Illumina MiSeq	PRJNA557906	NC_044185.1	PRJNA299862<br>PRJNA487526	[19,25,27,31]
Nepenthes spp. (Multi-species)	-	-	Illumina HiSeq;<br>Illumina MiSeq	PRJNA516491	-	PRJEB20488<br>PRJEB35235<br>PRJEB37794,PRJEB37797<br>GenBank: MH544206–MH544225	[29,54]
S. alata	3,162	-	PacBio Sequel,<br>Illumina HiSeq,<br>BioNano;<br>Roche 454 GS-FLX;<br>Illumina MiSeq	PRJNA604471<br>GenBank: JN665096–JN667881	-	PRJNA609445 (metatranscriptome)	[4]
S. psittacina	-	-	Roche 454 GS-FLX	-	-	PRJNA80051	[5]
S. purpurea	-	-	Roche 454 GS-FLX	-	-	PRJNA80053	[5]
U. gibba	100.7 (PacBio assembly)	152.1	Roche 454 GS-FLX;<br>Ion Torrent PGM;<br>PacBio RS;<br>Illumina HiSeq	PRJNA207602<br>PRJNA383049<br>PRJNA633566 (methylome)	NC_021449.1	PRJNA79917<br>PRJNA189698<br>PRJNA354080<br>PRJNA574143<br>PRJNA595351	[8,10,51]
U. reniformis	317.1	139.7	Illumina HiScanSQ,<br>Illumina MiSeq,<br>Ion Torrent Proton;<br>Roche 454 GS-FLX	PRJNA290588<br>PRJNA302107<br>PRJNA316304<br>PRJNA374515	NC_029719.2	PRJNA290588	[9,68]

- : not available; References can refer to the main manuscript.
